# Targeted Connectomic Neuromodulation of the Orbitofrontal Cortex To Treat Obsessive-Compulsive Disorder

**DOI:** 10.64898/2026.05.26.26354163

**Published:** 2026-05-28

**Authors:** Emma Anderson, Audrey Kist, Zachary D Simon, Jason Raj, Sneha Ray, Dani Astudillo, Natalie Becker, Tenzin Norbu, Starlette Khim, Dennis Lambert, John Alvarez, Kelly Kadlec, Anusha B Allawala, Alexandra Tremblay-McGaw, Jessica Verhein, Caroline A Racine, Pierre Naldec, Ahmad Alhourani, Keaton Piper, Joline M Fan, Doris D Wang, Ankit N Khambhati, Kristin K Sellers, Phillip A Starr, Leo P Sugrue, Edward F Chang, Andrew D Krystal, A Moses Lee

## Abstract

Pathological activity within frontal cortical circuits is common in many neuropsychiatric disorders, such as obsessive-compulsive disorder (OCD). We developed an invasive brain mapping protocol in which temporary electrodes are implanted in candidate sites to identify personalized stimulation targets that can acutely relieve OCD symptoms. We found that stimulation within segments of the anterior limb of the internal capsule (ALIC) focally suppressed the structurally and functionally connected region of prefrontal and cingulate cortex. By leveraging the topographic organization of the ALIC, we reversibly inactivated frontal cortical sites with ALIC stimulation to determine which cortical regions are necessary for sustaining OCD symptoms. Stimulation of ventral capsule (VC) near the globus pallidus within the ALIC was associated with suppression of lateral orbitofrontal cortex activity and acute and long-term improvements in OCD symptoms. These results provide a paradigm for leveraging ALIC topography to deliver targeted connectomic neuromodulation to frontal cortex to treat neuropsychiatric disorders.

## Full Text Availability

The license terms selected by the author(s) for this preprint version do not permit archiving in PMC. The full text is available from the preprint server.

